# 1224. Outcomes of Empirical Vancomycin De-escalation Based on Methicillin-Resistant *Staphylococcus aureus* Nares

**DOI:** 10.1093/ofid/ofad500.1064

**Published:** 2023-11-27

**Authors:** Jiachen Xu, Arthur Chan, Bethany A Wattengel, Kari A Mergenhagen

**Affiliations:** Veterans Affairs Western New York Healthcare System, Buffalo, New York; Veterans Affairs Western New York Healthcare System, Buffalo, New York; VA WNY Healthcare System, Buffalo, NY; VA WNY Healthcare System, Buffalo, NY

## Abstract

**Background:**

Vancomycin is a first-line therapy for methicillin-resistant Staphylococcus aureus (MRSA) infections. Studies found that vancomycin is associated with a higher incidence of nephrotoxicity, even within therapeutic range. MRSA nares screening allows for rapid identification of MRSA carriers. It has been proposed that MRSA nares screening may improve patient care by avoiding vancomycin. Clinical data showing the impact of de-escalation based on MRSA nares is sparse. The objective of this study is to examine the incidence of nephrotoxicity, length of stay and clinical outcomes in those who were empirically deescalated vs. those who were not.

**Methods:**

This was a retrospective cohort study conducted across Veterans Affairs (VA) medical centers nationwide from 1 January 2018 to 1 August 2022. In addition to baseline patient characteristics, MRSA nares results on admission and vancomycin usage were obtained. Patients were stratified based on the timing of vancomycin de-escalation. Primary outcome was development of acute kidney injury, defined as a 50% increase in serum creatinine or increases in SCr by 0.3 mg/dL. Secondary outcomes included hospital length of stay and clinical course of empirical de-escalation. Odds ratios were generated from a multivariate logistic regression of significant factors.

**Results:**

This cohort yielded 49,340 cases across the nation. Vancomycin was de-escalated after 1 dose in 7196 cases (14.58%) after MRSA nares results being documented in the electronic health record. AKI rates were lower with the early de-escalation (377/7196[5.24%] vs 3185/42144 [7.56%], P< 0.0001). In multivariate analysis, incidence of nephrotoxicity was reduced by 30% (OR = 0.686; CI 0.612-0.767). Additionally, the mean hospital length of stay was reduced (8.5 vs 12 days).

**Conclusion:**

De-escalation of vancomycin based on MRSA nares screening result can reduce the risk of nephrotoxicity and decrease hospital length of stay.

**Disclosures:**

**All Authors**: No reported disclosures

